# Rehabilitation Approaches and Strategies in the Management of Adult Patients Following Pelvic Fractures: Protocol for a Scoping Review

**DOI:** 10.2196/38884

**Published:** 2023-05-03

**Authors:** Ntombenkosi Appears Sobantu, Muziwakhe Daniel Tshabalala, Verusia Chetty

**Affiliations:** 1 Department of Physiotherapy University of KwaZulu-Natal Westville Campus Durban South Africa; 2 Department of Physiotherapy Sefako Makgatho Health Sciences University Pretoria South Africa

**Keywords:** rehabilitation approaches, rehabilitation strategies, adult patients, pelvic fractures, polytrauma, integrated health care, early intervention

## Abstract

**Background:**

Pelvic fractures can lead to disability and a poor health-related quality of life, thereby contributing to the burden of disease in South Africa. Rehabilitation plays an important role in improving the functional outcomes in patients with pelvic fractures. However, there is limited published research that presents optimal interventions and guidelines to improve outcomes in affected individuals.

**Objective:**

The purpose of this study is to examine and map the range of and gaps in rehabilitation approaches and strategies used by health care professionals globally in the management of adult patients with pelvic fractures.

**Methods:**

The synthesis of evidence will follow the framework outlined by Arksey and O’Malley and supported by the Joanna Briggs Institute. The identification of research questions; the identification of relevant studies; the selection of eligible studies; charting data; collating, summarizing, and reporting of the results; and consultation with relevant stakeholders will be undertaken. Peer-reviewed articles written in English; from quantitative, qualitative, and mixed methods studies; and searched through Google Scholar, MEDLINE, PubMed, and Cochrane Library will be considered. Studies eligible for selection will be full-text articles written in English about adult patients with pelvic fractures. Studies on children with pelvic fractures and on interventions following pathological pelvic fractures as well as opinion papers and commentaries will be excluded from the study. Rayyan software will be used for title and abstract screening to determine inclusion in the study and to improve collaboration between the reviewers. The Mixed Methods Appraisal Tool (version 2018) will be used to appraise the quality of the studies.

**Results:**

This protocol will guide a scoping review to examine and map the range of and gaps in rehabilitation approaches and strategies used by health care professionals globally in the management of adult patients with pelvic fractures, irrespective of level of care. Impairments, activity limitations, and participation restrictions in patients with pelvic fractures will be highlighted, which will give an indication of the rehabilitation needs of the affected individuals. Results of this review might provide evidence for health care professionals, policy makers, and scholars to aid rehabilitative care and further integration of patients into health care systems and community.

**Conclusions:**

The rehabilitation needs of patients with pelvic fractures will be drawn from this review and will be presented in a flow diagram. Rehabilitation approaches and strategies in the management of patients with pelvic fractures will be identified to guide health care professionals in the promotion of quality health care for these patients.

**Trial Registration:**

OSF Registries osf.io/k6eg8; https://osf.io/k6eg8

**International Registered Report Identifier (IRRID):**

PRR1-10.2196/38884

## Introduction

Pelvic fractures are mainly due to trauma [1] and can lead to disability and a poor health-related quality of life, thereby contributing to the burden of disease in South Africa [1]. The burden of disease hinders development and delays the attainment of sustainable development goals. Pelvic fractures may be complex injuries due to their association with polytrauma as a result of high-velocity impact [2]. The majority of individuals in India and South Africa who sustain pelvic fractures, secondary to motor vehicle accidents, are young male adults from both low and middle socioeconomic classes [3-5]. However, pelvic fractures in older adults are commonly associated with low-velocity impact in the United Kingdom [2].

The rate of survival of patients with pelvic fractures has increased significantly [6]. However, patients present with impairments and disabilities that affect their participation in the activities of daily living in their homes, society, and the community at large. Pelvic fractures present a challenge to the trauma and orthopedic surgeons due to their complexity [2]. They are further complicated by the associated injuries, which can lead to poor outcomes following management [3]. Poor outcomes were reported by patients following the management of pelvic fractures between 2008 and 2013 from different academic hospitals in Tshwane, South Africa (Sobantu, Skaal, and Tshabalala, unpublished data, 2017).

The aforementioned study explored the perspectives of patients on their health-related quality of life, following management of their pelvic fractures. Some of the challenges that were reported by the participants were poor mobility, chronic pain, lack of vitality, sexual dysfunction, and urinary and bowel incontinence. These challenges affected their day-to-day functioning at home, in society, in their communities, and at their workplaces. All these disabling outcomes resulted in low levels of physical health; psychological strain; poor social relations; and loss of independence in patients with pelvic fractures, regardless of the type of fracture. Pelvic fracture malunion leads to permanent disability in more than 65% of patients [7]. Some of the limitations in patients with pelvic fractures include gait abnormalities and chronic pelvic and back pain [8].

The increased survival rate of patients with pelvic fractures has been attributed to improved multidisciplinary health care management [9]. However, there is limited published research that presents optimal interventions and guidelines to improve outcomes in affected individuals. Patients with pelvic fractures commence their rehabilitation programs very late. Some patients even get discharged from hospitals before receiving rehabilitation. Health care professionals focus on the impairments that patients present with whilst hospitalized. Activity limitations and participation restrictions, which are more noticeable once the patient has been discharged, are rarely addressed. There is limited use of outcome measurement tools in these patients, especially by the rehabilitation team, to guide their progress.

Early appropriate care optimizes health care and results in fewer complications in patients following pelvic fractures [10]. The provision of early appropriate care has been a challenge in most health institutions due to limited human resources and few hospital beds. Few hospital beds lead to the early discharge of patients. These challenges lead to some patients not receiving optimum, integrated health care, including rehabilitation. Loss of continuity in health care can occur due to patients being discharged without home programs or relevant referrals for outpatient rehabilitation. Loss of continuity leads to an increase in long-term disabilities in these patients.

Even though pelvic fractures are a challenge to manage due to their complexity, no standardized comprehensive guidelines have been published to address the problems associated with them [11]. Guidelines and models are important in providing clear strategies to health care professionals involved in the management and rehabilitation of patients with pelvic fractures. Guidelines and models can assist in guiding the rehabilitation of patients with pelvic fractures within the biopsychosocial model of care, as guided by the International Classification of Functioning, Disability and Health framework [12]. An integrative rehabilitation approach will improve patient health outcomes and reduce the incidence of residual disabilities. Physiotherapists play a major role in the rehabilitation of patients with pelvic fractures. They work toward reducing pain, improving joint mobility, strengthening muscles, addressing pelvic dysfunction, and promoting function. The aforementioned aims are achieved by using therapeutic exercises, manual therapy, and electrotherapy.

Therefore, the aim of this scoping review is to examine and map the range of and gaps in rehabilitation approaches and strategies used by health care professionals globally in the management of both in- and outpatients with pelvic fractures, irrespective of level of care.

## Methods

### Overview

**Figure 1:**
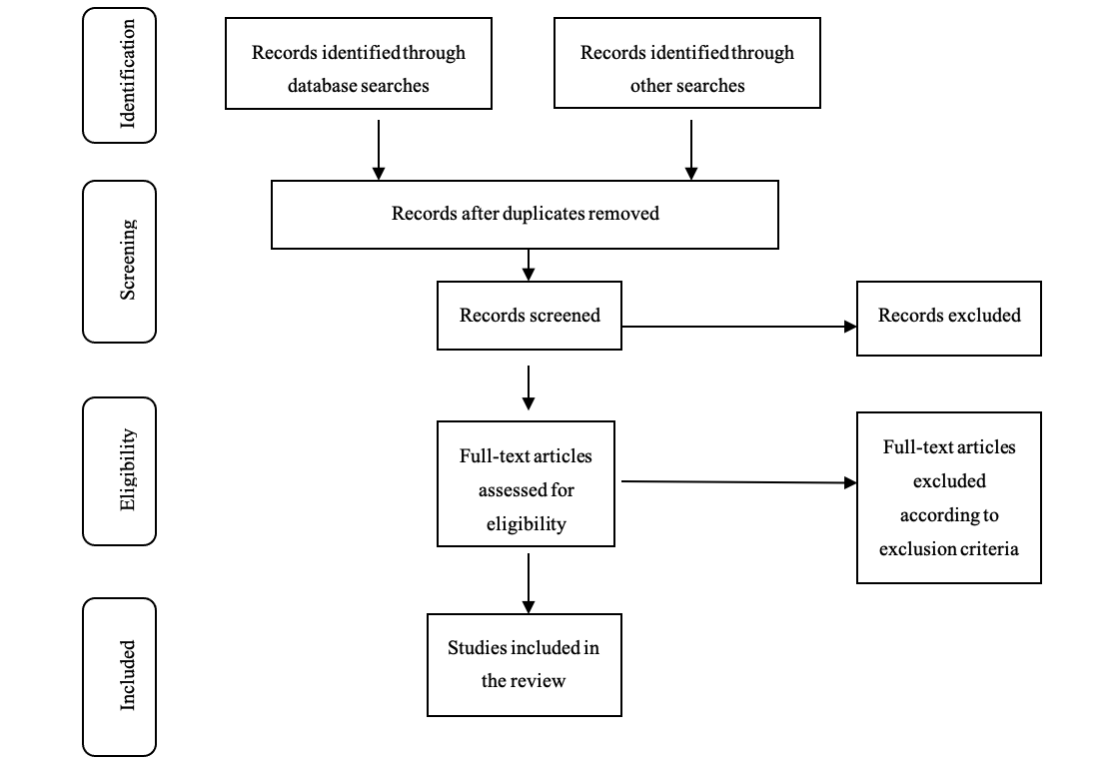
PRISMA-ScR (Preferred Reporting Items for Systematic Reviews and Meta-Analyses extension for Scoping Reviews) flow diagram [13].

This protocol has been registered with the Open Science Framework (osf.io/f9w3z). This study protocol follows the reporting guidelines provided in the PRISMA-P (Preferred Reporting Items for Systematic Review and Meta-Analysis Protocols ) statement [12] and the PRISMA-ScR (Preferred Reporting Items for Systematic Reviews and Meta-Analyses extension for Scoping Reviews; Figure 1; Multimedia Appendix 1) [13]. Literature on rehabilitation approaches and strategies in the management of adult patients following pelvic fractures will be reviewed. This review will be guided by Arksey and O’Malley’s methodological framework [14]. The framework identifies 6 stages that must be considered when developing a scoping review. The 6 stages are as follows: identification of the research question; identification of relevant studies; selection of eligible studies; charting the data; collating, summarizing, and reporting the results; and consulting with relevant stakeholders. The stakeholders include the orthopedic surgeon, urologist, psychologist, physiotherapist, dietician, orthopedic nurse, occupational therapist, social worker, orthotist, and prosthetist, and general or trauma surgeon. Results of the scoping review will be sent by email to the stakeholders for critiquing and reflecting on the evidence. Virtual meetings may be arranged as a follow-up to emails should the need arise. Stakeholders will have an opportunity to provide possible solutions to develop a fit-for-purpose interprofessional education and collaborative practice model. The purpose of including the stakeholders is to ensure that the best quality of evidence is collated and implemented.

### Identifying the Research Question

The research question underpinning the review is “What rehabilitation approaches and strategies have been integrated in the management of adult patients with pelvic fractures?”

Subquestions that will guide the review are as follows:

- What evidence exists on the rehabilitation approaches and strategies for skeletal stabilization of pelvic fractures, globally?
- What evidence exists on the rehabilitation approaches and strategies for associated injuries (eg, rectal and urogenital, chest and abdomen, and other musculoskeletal systems) in adult patients with pelvic fractures?
- What evidence exists on rehabilitation interventions for impairments (eg, pain, joint mobility and function, as well as emotional and psychological challenges) experienced by adult patients following pelvic fractures?
- What evidence exists on rehabilitation interventions for functional disabilities, including mobility, urinary and bowel incontinence, and sexual dysfunction experienced by adult patients following pelvic fractures?

### Information Sources and Search Strategy

Identification of studies relevant to this review will be achieved in accordance with the Joanna Briggs Institute search strategy [15]. A search for relevant literature will be conducted on the following electronic databases from 2002 to 2022: Google Scholar, MEDLINE, PubMed, and Cochrane Library. Keywords relevant to each search parameter will be separated by the Boolean terms “AND,” “OR,” and “NOT.” A draft search strategy in PubMed is documented in Multimedia Appendix 2. The reference section of the selected studies will also be explored to identify further, potentially relevant studies that could have been missed in the initial search.

A pilot search using keywords to determine the feasibility of the study will be conducted (Multimedia Appendix 2). The search strategy will be adjusted for each database.

### Eligibility of Research Studies

The study will incorporate the Population, Concept, and Context (PCC) model to align the study selection with the research question [15]. The Population, Concept, and Context model is a well-formulated model that improves scientific rigor for scoping review questions. Studies will have to include globally published articles on adult patients with pelvic fractures focused on rehabilitation approaches and strategies used during management. Rehabilitation approaches and strategies used to address skeletal stability, associated injuries, as well as impairments and disabilities in adult patients with pelvic fractures will be included. Articles on the functional outcome measurements used, home programs, and advice and education that can be given to adult patients with pelvic fractures will also be included. Peer-reviewed journal articles written in English will be included. Quantitative, qualitative, and mixed methods studies will also be included. All health care systems, including primary, secondary, and tertiary systems, will be included. Titles, abstracts, and full texts will be screened by 2 reviewers (MDT and NAS). Discrepancies and disagreements will be resolved by discussions between the 2 reviewers and a third reviewer (VC). Rayyan software will be used for title and abstract screening to determine inclusion in the study and to improve collaboration between the reviewers [16,17].

### Inclusion and Exclusion Criteria

The Inclusion and exclusion criteria for studies eligible for selection are presented in Textbox 1.

**Inclusion and exclusion criteria for study selection.**

**Inclusion criteria**

- Articles about adults (aged ≥18 years) with pelvic fractures
- Global articles on rehabilitation approaches and strategies used during management
- Full-text studies
- Articles written in English

**Exclusion criteria**

- Articles on children with pelvic fractures
- Articles on interventions following pathological pelvic fractures
- Opinion papers
- Commentaries

### Charting of the Data

A data charting template (Textbox 2) will be used to extract and capture information from studies through each phase of the review. The information will be about rehabilitation approaches and strategies used by the health care team in the management of patients with pelvic fractures. The chart will be continually and regularly updated by the reviewers through the scoping process to capture all possible results for the research question. Two reviewers will chart the data independently, discussing the results and updating the chart.

**Data charting form.**

**Data charting information**

- Author(s)
- Year of publication
- Origin or country of origin (where the study was published or conducted)
- Aims or purpose
- Study population
- Sample size (if applicable)
- Methodology or methods
- Intervention
- Details of interventions (if applicable)
- Outcomes (if applicable)
- Details of these of outcomes (eg, how measured, if applicable)
- Key findings that relate to the scoping review question(s)
- Conclusions that relate to the scoping review questions

### Collating, Summarizing, and Reporting Results

The data extracted from the studies will be guided by the research questions and subquestions. This will include evidence that exists on the rehabilitation approaches and strategies for each impairment as well as activity limitations and participation restrictions in adult patients with pelvic fractures. The stages of the scoping review promote transparency and allow reproducibility of the study by reducing the risk of bias and data duplication [14,18]. The extracted data will be summarized in the final write-up using a thematic analysis with flexibility in the capturing of data, as in the scoping review [18]. The results will be interpreted and described according to the research question and subquestions.

### Quality Appraisal and Bias

Quality appraisal will be undertaken in this scoping review to ensure that strong evidence is collected, which will help to create a trustworthy guide for follow-up research projects. Performance of quality appraisal is informed by a study by Daudt et al [19] who underlined the importance of assessing the quality of studies and conducting a trial of the method before starting with the charting process, if the research is to be used to inform policy makers. The Mixed Methods Appraisal Tool (MMAT; version 2018) will be used to assess the quality of the studies from the search strategy, described above [19]. Moreover, MMAT will be used to assess and report bias from quantitative, qualitative, and mixed methods studies [20]. MMAT is suitable as the study excludes articles such as commentaries and opinion papers. The process requires critical appraisal, and therefore, 3 reviewers will be involved in the appraisal process. One reviewer has experience in MMAT application and will add rigor to the appraisal. The MMAT method allows for the inclusion of qualitative studies, quantitative randomized controlled trials, quantitative nonrandomized trials, and quantitative descriptive and mixed methods studies. Methodological quality criteria will be captured as per the MMAT by 2 reviewers, with the third reviewer overseeing the process.

## Results

This protocol will guide a scoping review to examine and map the range of and gaps in rehabilitation approaches and strategies used by health care professionals globally in the management of both in- and outpatients with pelvic fractures, irrespective of level of care. Impairments, activity limitations and participation restrictions in patients with pelvic fractures will be highlighted, which will give an indication of the rehabilitation needs of the affected individuals. Results of this review might provide evidence for health care professionals, policy makers, and scholars to aid rehabilitative care and further integration of patients into health care systems and community.

## Discussion

### Expected Outcomes

These guidelines will inform the scoping review of examining the range of and gaps in the literature on the rehabilitation approaches and strategies for the management of patients with pelvic fractures. The rehabilitation needs of patients with pelvic fractures will also be drawn from the results of this review and presented in a flow diagram. This study might be used to inform health care professionals of the important aspects to be included in the assessment, management, and rehabilitation of patients with pelvic fractures. The results of this study might also be used to improve the physiotherapy curriculum. Approaches to health care management by health care professionals might be guided by the findings of this review. Health care professionals might be aware of the impact of pelvic fractures on the physical, mental, psychological, and socioeconomic status of the affected individuals. Health care professionals might be able to draw up relevant and patient-centered ward and home programs that can promote health-related quality of life in patients who had sustained pelvic fractures.

This scoping review is the first phase of a study that seeks to develop an interprofessional model of care for patients with pelvic fractures. It is hoped that this review will create awareness of the health care needs of patients with pelvic fractures. This awareness might prompt the health care professionals to set goals that will address most of the patients’ needs. The rehabilitation approaches and strategies used by the health care professionals globally as well as gaps in the literature will be identified [21]. This information will be crucial in informing the envisaged model with the best practical evidence, especially pertaining to physiotherapy practice, for patients with pelvic fractures.

The findings of the study will also provide evidence for health care policy makers to assist the stakeholders in addressing the needs of this population by rendering quality health care.

### Conclusions

This scoping review protocol outlines steps that will guide the scoping review. This review will also identify and map studies that indicate the rehabilitation approaches and strategies in the management of patients with pelvic fractures. The results of this study may promote circulation of information and knowledge among health care professionals at various levels of health care.

### Limitations

Some articles might not be available in the databases that will be included in the study. Literature might also be limited since only articles written in English and available in full text will be considered for the study.

## Appendix

Multimedia Appendix 1 Data search strategy.

Multimedia Appendix 2 PRISMA-ScR (Preferred Reporting Items for Systematic Reviews and Meta-Analyses extension for Scoping Reviews) fillable checklist.

## Abbreviations

MMAT: Mixed Methods Appraisal Tool
PRISMA-P: Preferred Reporting Items for Systematic Review and Meta-Analysis Protocols
PRISMA-ScR: Preferred Reporting Items for Systematic Reviews and Meta-Analyses extension for Scoping Reviews

## Data Availability

Articles that will be included in the study will be deposited into the university repository and will be made available when necessary.
