# Supplementary material for: Rehabilitation Approaches and Strategies in the Management of Adult Patients Following Pelvic Fractures: Protocol for a Scoping Review
Source: JMIR Res Protoc. 2023 May 3;12:e38884. doi: 10.2196/38884 (PMC10193220; doi:10.2196/38884)
Supplement: Multimedia Appendix 2 [file resprot_v12i1e38884_app2.docx]

**DRAFT SEARCH STRATEGY - Rehabilitation approaches and strategies in the management of adult patients following pelvic fractures: a scoping review protocol**

| **Keywords Searched** | **Data Base** | **Date searched** |
| --- | --- | --- |
| ("patient" OR "patients" OR "inpatient" OR "inpatients" OR "outpatient" OR "outpatients" OR "hospitalized" OR "hospitalization" OR "institution" OR "institutionalized" OR "adult" OR "adults" OR "young adult" OR "middle age") AND (("rehabilitation" OR "therapy" OR "therapies" OR "management" OR "strategy" OR "approach" OR "approaches" OR "intervention" OR "interventions" OR "patient care" OR "after care" OR "function" OR "functioning" OR "mobile" OR "ambulation" OR "surgery" OR "surgeries" OR "health" OR "health services" OR "care" OR "care program")) AND ((("pelvic fracture" OR "pelvic fractures" OR "fracture" OR "fractures" OR "pelvis" OR "pelvic" OR "pelvic region"))) | PubMed  197 166 | 16/11/2022 |
| "rehabilitation approaches" OR "rehabilitation strategies" OR "rehabilitation" AND "adult patients" OR "adult patient" AND "pelvic fracture" OR "pelvic fractures" | PubMed  1890 | 17/11/2022 |
| (rehabilitation or therapy or treatment or intervention or physiotherapy or physical therapy or counselling) AND (adult patients or adults) AND (pelvic fracture or pelvis fracture) | Ebscohost  22 184 | 16/11/2022 |
